# Efficient regulatory approval of two novel HIV prevention interventions in a resource-limited setting: experiences from Zimbabwe

**DOI:** 10.3389/frph.2023.1279124

**Published:** 2023-11-14

**Authors:** Caroline Murombedzi, Libert Chirinda, Gift T. Chareka, Z. Mike Chirenje, Nyaradzo M. Mgodi

**Affiliations:** ^1^ Faculty of Medicine and Health Sciences, University of Zimbabwe, Clinical Trial Research Centre, Harare, Zimbabwe; ^2^ Pharmacovigilance and Clinical Trials Division, Medicines Control Authority of Zimbabwe, Harare, Zimbabwe; ^3^ School of Medicine, University of California San Francisco, San Francisco, CA, United States

**Keywords:** HIV, pre-exposure prophylaxis, dapivirine vaginal ring, long-acting injectable cabotegravir, regulatory approval, Zimbabwe

## Abstract

The global burden of HIV remains unacceptably high despite significant progress made in HIV treatment and prevention. There is an urgent need to scale up the comprehensive HIV prevention strategies that include pre-exposure prophylaxis (PrEP). Oral PrEP is highly effective in preventing HIV acquisition when taken regularly, but this remains a challenge for some at-risk individuals. Therefore, there is a need for other HIV prevention options. The dapivirine vaginal ring (DVR) and long-acting injectable cabotegravir (CAB-LA) are novel biomedical interventions that are safe and efficacious for HIV pre-exposure prophylaxis, as demonstrated in recently completed clinical trials. Timely roll-out and scalability of efficacious interventions depend on the registration process with the national medicine regulatory authorities (NMRAs). The Medicines Control Authority of Zimbabwe (MCAZ) was the first NMRA globally to approve the DVR in July 2021 and the first in Africa to approve CAB-LA for HIV prevention in July 2022. The regulatory review process for DVR and CAB-LA by MCAZ took 4.5 and 5.5 months, respectively. This efficient review process of the two interventions by MCAZ, a regulatory body in a resource-limited setting, provides important lessons to shorten timelines between the completion of the clinical development process and the registration of essential medicines.

## 1. Introduction

Remarkable progress has been made in HIV treatment and prevention in the last two to three decades. Since the 1990s, HIV incidence has been reduced by 90% in Zimbabwe, 45% in South Africa, and 44% in Kenya (1). Overall, AIDS-related deaths in Eastern and Southern Africa have decreased by 58%, and the number of new HIV infections has also decreased by 57% since 2010 (2). Notably, in sub-Saharan Africa (SSA), a 39% decrease in HIV incidence was reported among young women aged 15–24 years between 2010 and 2020 (1). Despite this significant progress, HIV/AIDS is still a major global public health threat, with 1.3 million new HIV infections and 630,000 AIDS-related deaths per year. In 2022, AIDS claimed a life every minute, and 4,000 adolescent girls and young women were diagnosed with HIV each week (2). Of all the new HIV infections in SSA in 2022, 63% were among women and girls (2). Zimbabwe is at the epicenter of the global HIV pandemic, with an HIV prevalence of 12.9 % among adults, 15.3% among women, and 10.2% among men aged 15–49 years (3). The annual incidence of HIV was 0.45% among Zimbabwean adults aged 15–49 years, 0.67% among women and 0.23% among men. An estimated 31,000 new HIV cases occurred among Zimbabwean adults in 2020 (3). With the current rate of new HIV infections, the world will not meet the Global AIDS Strategy (2021–2026) target of fewer than 370,000 annual incident HIV infections by 2025 (1).

There is an urgent need to scale up comprehensive HIV prevention strategies that include the provision of pre-exposure prophylaxis (PrEP) options of oral Truvada, the dapivirine vaginal ring (DVR), and long-acting injectable cabotegravir (CAB-LA). This requires regulatory approval of the PrEP products before they can be made available. DVR and CAB-LA have not been approved in many countries yet, which limits the available PrEP options. Their prompt and easy regulatory approval has the potential to greatly decrease the rate of new HIV infections, especially in SSA, where the disease burden is the highest. Many countries in SSA have limited resources to conduct regulatory reviews of new drug applications. Countries with limited resources have reduced workforce and materials for efficient drug regulation. As a result, drug regulatory approvals in these countries are prolonged, and access to essential medicines is delayed. In this article, we aimed to describe how the National Medicine Regulatory Authority (NMRA) in Zimbabwe, a resource-limited country in Southern Africa, was able to approve DVR and CAB-LA in a short period.

## 2. Drug approval process: from clinical trials to scale-up

Proven safety, efficacy, and acceptability of new biomedical interventions from clinical trials do not guarantee access but are merely one step in ensuring the availability of the new agents. PrEP is the use of antiretroviral drugs to prevent HIV acquisition. Before being made available to the public, PrEP agents must be registered for use by the drug regulatory authority in the country where they will be distributed. The products must be scalable to reach people who could benefit from them most promptly.

Many countries took years to approve Truvada (emtricitabine/tenofovir disoproxil fumarate). Truvada was initially submitted to the Medicines Control Authority of Zimbabwe (MCAZ) in May 2006 for registration as an antiretroviral drug for HIV treatment and received approval in April 2009. The process took almost 3 years. Oral PrEP with Truvada was first approved in the United States by the US Food and Drug Administration (FDA) in 2012. The World Health Organization (WHO) recommended offering Truvada as part of a comprehensive HIV prevention package to individuals at substantial risk of HIV infection in 2015 (4).

Oral PrEP is highly effective in preventing HIV acquisition when taken regularly, but this remains a challenge for some at-risk individuals, especially women in SSA, for a variety of reasons such as pill burden, stigma, fear of violence from partners, religious beliefs, and dissatisfaction with the PrEP delivery services (5, 6). Therefore, there is a need for discrete, safe, efficacious, and acceptable longer-acting HIV prevention options, such as the DVR and CAB-LA.

### 2.1. DVR regulatory pathway

The DVR is a discrete, flexible silicone ring that is inserted into the vagina and works by releasing the non-nucleoside reverse transcriptase inhibitor (NNRTI) dapivirine, an antiretroviral drug, slowly over a month (7, 8). The DVR, developed by the International Partnership for Microbicides (IPM), has been extensively evaluated across several phase I–III trials (6, 9). The IPM, together with the Microbicides Trials Network (MTN), conducted two large phase III trials, namely, the MTN-020/ASPIRE and The Ring study, assessing the safety, efficacy, and acceptability of the DVR in 4,500 women from Malawi, South Africa, Uganda, and Zimbabwe. The DVR was shown to reduce the risk of HIV-1 acquisition via vaginal sex by about 30% among cis-gender women, and further analysis demonstrated an HIV-1 risk reduction of 50% or more for those who used the ring most regularly (5, 8, 9). Modeling data from two subsequent open-label studies (i.e., MTN-025/HOPE and DREAM) suggested a greater HIV-1 risk reduction of 63% or more for those who used the ring most regularly across both studies (8, 10). The DVR showed a strong safety profile and no evidence of NNRTI resistance among women who seroconverted across the four studies (8–11). The clinical trials were supported by qualitative work with women and their sexual partners to understand end-user preferences and concerns. In Zimbabwe, ASPIRE and HOPE were conducted at three clinical research sites at the University of Zimbabwe Clinical Trials Research Centre (UZ-CTRC) (8, 9).

Following the successful phase I–III clinical trials, the IPM applied to the European Medicines Agency (EMA) to review the DVR under EU-M4all in 2017. On 24 July 2020, using data from the two phase III trials, the EMA issued a positive scientific opinion on the DVR (12), a piece of evidence that the product should be authorized or approved for use. The DVR subsequently received WHO prequalification in November 2020. In January 2021, the WHO recommended offering the DVR as a safe and effective additional prevention choice for women at significant risk of HIV infection as part of a package of prevention options. The WHO added the DVR to the WHO list of prequalified (essential) medicines after this process (13).

**Figure 1:**
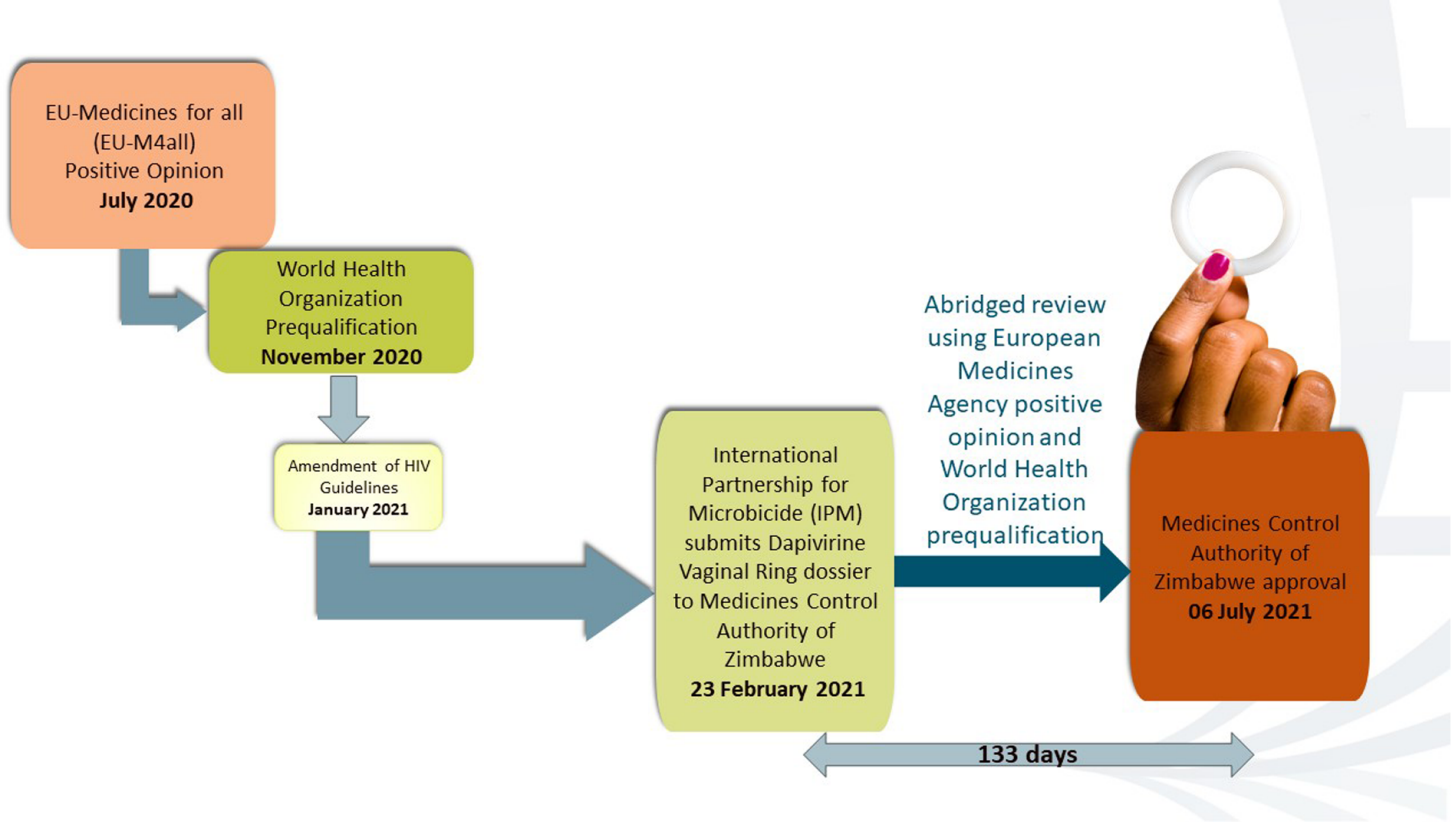
DVR regulatory pathway. Adapted from AIDS Vaccine Advocacy Coalition (AVAC) (12).

After WHO’s prequalification, on 23 February 2021, the IPM submitted the DVR dossier to MCAZ, NMRA in Zimbabwe, for review. The MCAZ Registration Committee did not conduct a full review. Instead, they used a reliance approach in line with the MCAZ Reliance guidelines (14). These guidelines allow the Registration Committee to rely on part or all of the decisions made by reference regulators. This is known as an abridged review (14). An abridged review enabled the process to be concluded in a shorter timeline than the normal review pathway. The positive scientific opinion from the EMA, the WHO prequalification, and the prior “regulatory knowledge” of MCAZ about the product from the clinical trial approval and oversight stages of the Zimbabwe UZ-CTRC sites enabled the MCAZ to conduct an expeditious but rigorous review. On 6 July 2021, 133 days after submission by IPM, MCAZ approved the ring for registration, and Zimbabwe became the first country globally to register the DVR (15, 16). The regulatory process for DVR in Zimbabwe is shown in Figure 1 adapted from AVAC (12).

### 2.2. CAB-LA regulatory pathway

Cabotegravir is a second-generation integrase strand transfer inhibitor (INSTI) antiretroviral drug with a high resistance barrier. The long-acting formulation, which is administered as an injection 1 month apart for the first 2 months and then every 2 months after that, was developed by ViiV Healthcare, a division of GlaxoSmithKline plc (GSK) (5). Two international phase IIb/III multicenter, randomized, double-blind, active-controlled studies, namely, HPTN 083 and HPTN 084, demonstrated the superiority of CAB-LA to Truvada for HIV prevention in individuals at high risk of HIV acquisition through vaginal and anal intercourse (6, 17). Compared to Truvada, the participants on CAB-LA experienced a 69% and a 90% lower rate of HIV acquisition in HPTN 083 and HPTN 084, respectively (6, 17). HPTN 084 enrolled 3,200 cis-gender women at 20 clinical research sites in Botswana, Eswatini, Kenya, Malawi, South Africa, Uganda, and Zimbabwe. The five sites of the University of Zimbabwe Clinical Trials Research Centre contributed almost 25% of the sample size (6).

**Figure 2:**
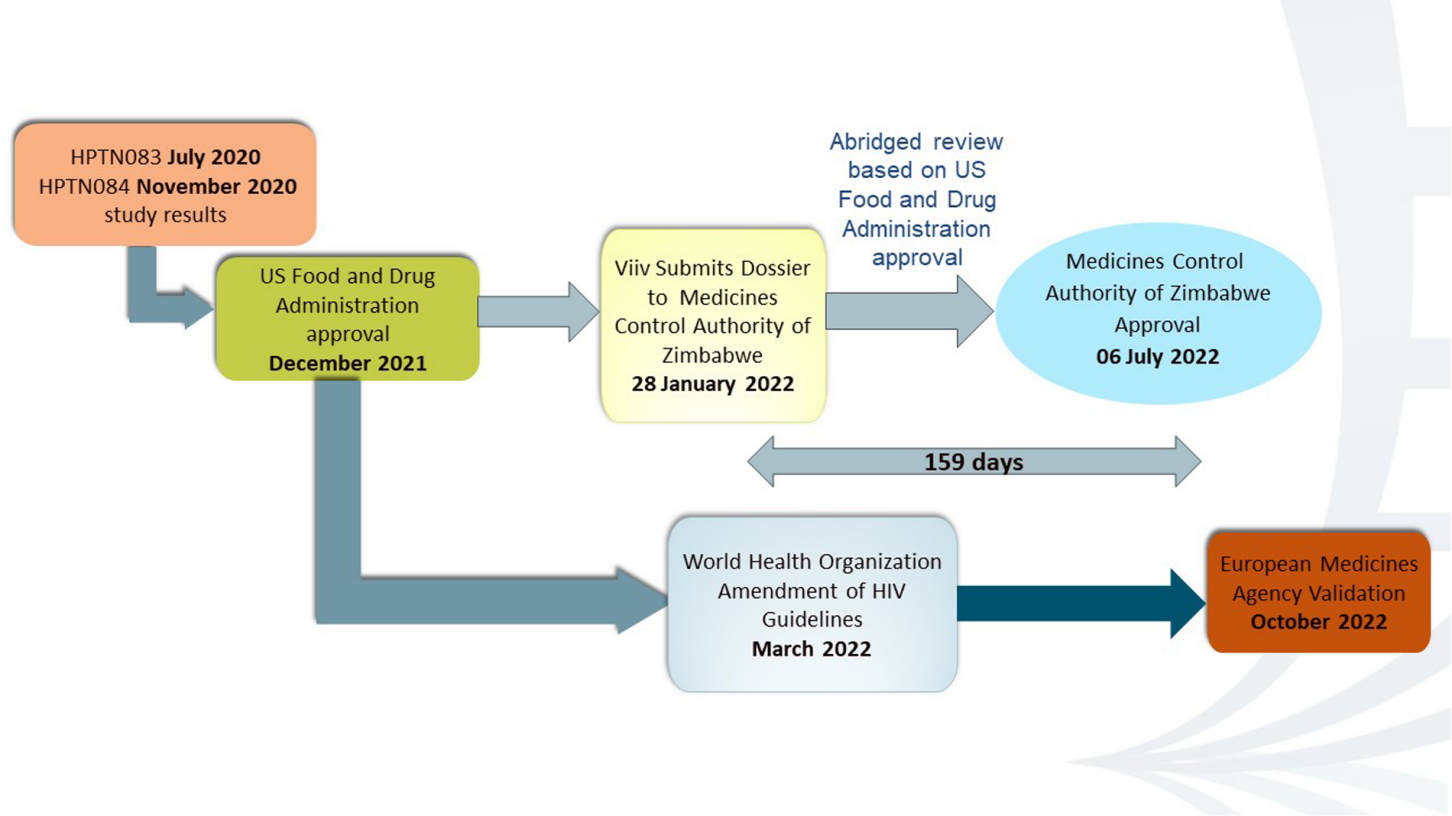
CAB-LA regulatory pathway.

Data from HPTN 083 and HPTN 084 supported the FDA regulatory approval for CAB-LA for PrEP on December 20, 2021 (18). In March 2022, the WHO recommended offering CAB-LA as an additional HIV prevention option. The EMA validated the marketing authorization application (MAA) of ViiV Healthcare for cabotegravir long-acting injectable for PrEP to reduce the risk of sexually acquired HIV-1 in October 2022 (19). This meant that the EMA had accepted the application and would begin the formal scientific review process. On January 28, 2022, GSK, the parent company of ViiV Healthcare, applied for registration of CAB-LA to the MCAZ. The FDA is one of the reference regulatory authorities (RRAs) that the MCAZ considers for reliance on the regulation of medicines. Being an innovative product from a “Stringent Regulatory Authority (SRA),” CAB-LA was eligible for the expedited review pathway. The MCAZ dossier assessors utilized the expedited review pathway before tabling for consideration during the monthly Registration Committee meetings. On July 6, 2022, Zimbabwe was the first African country and the first low- and middle-income country to approve CAB-LA for HIV prevention after the United States, as depicted in Figure 2 (20).

## 3. Capacity and expertise that strengthened the MCAZ

It took 133 days in 2021 to register the ring and 159 days in 2022 to register CAB-LA in Zimbabwe. Prior to that, between 2003 and 2015, it took 516–1,673 median days to register a drug in Zimbabwe. Truvada took 1,045 days to be registered in Zimbabwe in 2009. In 2019, the median overall approval time was reduced to 473 days (15.8 months) for new active substances, which is comparable to mature and better-resourced regulatory agencies (21). Several strategies provided MCAZ with the capacity and expertise to approve DVR and CAB-LA for registration in a timely manner. The MCAZ used the abridged review to approve DVR and CAB-LA in Zimbabwe because the two products had been previously approved by at least one reference authority. MCAZ considers some SRAs as reference authorities because they have strict standards for safety, efficacy, and quality. These SRAs include the EMA, US FDA, Australian Therapeutic Goods Administration, Health Canada, and Japanese Pharmaceuticals and Medical Devices Agency (21, 22). Other processes utilized by the MCAZ are as follows:

(i) Full review for products not approved by any reference agencies
(ii) Verification review for WHO-prequalified products through the WHO Collaborative Medicines Registration Procedure (CRP)
(iii) Mutual Recognition
(iv) Joint assessment and work-sharing [ZaZiBoNa initiative of the Southern African Development Community (SADC) harmonization initiative]
(v) Unilateral recognition (14, 22)

### 3.1. Pharmacovigilance and Clinical Trials division and Evaluations and Registration division

A major strength of the MCAZ is the seamless collaboration between the Pharmacovigilance and Clinical Trials (PVCT) division, which regulates and monitors clinical trials conducted in Zimbabwe, and the Evaluations and Registration (EVR) division, which assesses applications for registration of medicines in Zimbabwe. Prior to implementation, MTN-020/ASPIRE, MTN-025-HOPE, and HPTN 084 were approved by MCAZ through the PVCT, which worked closely with the EVR division. The latter provided an expert review of the information on chemistry, manufacturing, and controls (CMC) for the DVR and CAB-LA. This is one of the strengths that enabled a faster review of the two products when they were eventually submitted for registration in Zimbabwe; some of the key aspects of the products had already been evaluated and approved by the MCAZ at the clinical trial stage. In addition, throughout trial implementation, the PVCT division regularly received and monitored adverse reaction reports from the three pivotal studies in Zimbabwe. A specialized committee of the PVCT routinely conducted a causality assessment of adverse reaction reports to determine if an event was related to the DVR or CAB-LA (14, 21). This ongoing assessment further expedited the approval process for the two products.

### 3.2. WHO prequalification

The WHO prequalification was instrumental in the expeditious review of DVR/CAB-LA in Zimbabwe. The WHO prequalification guaranteed the quality, safety, and efficacy of the two products, and this contributed to the approval of NMRA for registration. The WHO Prequalification Programme helped in the capacity building of MCAZ in regulatory processes and inspections. MCAZ staff participated in dossier assessments and inspections conducted by the WHO (21, 23). The WHO Collaborative Registration Procedure pilot, which started in 2012 with Zimbabwe and nine other countries, provided capacity building that enabled faster registration of prequalified medicines and will avoid duplication of work in developing countries.

### 3.3. The European Medicines Agency

Since 2004, the EMA, working together with WHO, has been providing scientific opinions on essential human medicines that are planned for distribution in markets outside of the European Union (EU) (24). The purpose is to make access to essential medicines in low and low-middle-income countries (LMICs) with limited regulatory capacity much easier for patients. This procedure is called EU-Medicines for all (EU-M4all) and was previously known as the Article 58 procedure. The procedure is a combination of the scientific review capabilities of the EMA with the expertise of the WHO and national regulators in the target countries on the local epidemiology and disease. This provides a unique development and assessment pathway. Under this procedure, the Committee for Medicinal Products for Human Use (CHMP) of the EMA reviews medicines and vaccines to similar high standards to medicines intended for use in Europe. After the evaluation, the EMA publishes its scientific opinion of the benefit–risk balance of the product, which aims to facilitate prequalification of the medicine by the WHO and registration in the intended countries (24). The positive opinion of the EMA on the DVR enabled the IPM application to be reviewed under the abridged review process by MCAZ and resulted in approval within 133 days.

### 3.4. SPaRCS project in Southern African countries

MCAZ was also part of the strengthening pharmacovigilance and regulatory capacities in four Southern African countries (SPaRCS) project with Eswatini, Namibia, and South Africa from April 2020 to October 2023. The inclusion of MCAZ in the SPaRCS project strengthened the regulatory capacities of the authority through a participatory learning approach. The project will potentially pave the way for harmonization of requirements and reliance in the region in regulatory issues involving clinical trials and pharmacovigilance (25), making approval of future biomedical products even more expeditious.

### 3.5. ZaZiBoNa initiative

Zimbabwe is a founding member, alongside Zambia, Botswana, and Namibia, of the SADC collaborative medicine registration initiative, namely, the ZaZiBoNa initiative, which was established in 2013 (21, 22, 26, 27). The initiative has contributed to MCAZ capacity building by providing a program for training and enabling collaboration with other regulatory authorities. The initiative also strengthened the capacity of MCAZ by reducing workload and time to registration and developing mutual trust and confidence in regulatory collaboration (26, 27). Harmonization of registration requirements and joint reviews were also shown to have contributed to reduced workload for both the pharmaceutical industry and the regulatory authorities in the ZaZiBoNa countries (26).

## 4. Discussion

The Zimbabwean NMRA, i.e., MCAZ, is a member of several regulatory partnerships, such as the WHO Collaborative Registration Procedure and ZaZiBoNa initiative of the SADC. These collaborations have strengthened the regulatory capacity of MCAZ. MCAZ uses different models to approve medicines, including the abridged review, where review time is shortened when the product is already approved by one reference authority. This review type enabled Zimbabwe to register DVR and CAB-LA in a shorter time than other drug regulatory authorities. NMRAs in the region and other LMICs should emulate MCAZ by establishing similar collaborations in their geographical regions and with well-resourced authorities in other parts of the world.

The establishment of a SADC regional medicines authority has been suggested by previous researchers and is highly recommended to streamline the registration process across the region (26). A regional medicines authority, such as the EMA that regulates and monitors medicines for the entire European Union, would mean one approval would simultaneously accelerate access in many countries. Similarly, the establishment of the African Medicines Agency (AMA), whose treaty-signing process is currently ongoing, is expected to strengthen the capacity of African countries to regulate medicines and related products, provide regulatory guidance, and harmonize medical regulation efforts on a continental level (28, 29).

MCAZ was designated a Regional Centre of Regulatory Excellence (RCORE) under the African Medicines Regulatory Harmonization (AMRH) Initiative of the African Union and the New Partnership for Africa's Development (NEPAD) Agency in 2014. RCORE offers training services in medicine registration, PVCT, and laboratory testing of medicines for new and experienced regulators from NMRAs, regulatory affairs personnel from the pharmaceutical industry, and academia to increase the regulatory workforce in Africa (28). Regulators from other LMICs should participate in these courses to learn from the best practices of MCAZ in medicine registration and other regulatory aspects.

High staff turnover and poor skill retention have been reported at MCAZ and other NMRAs in the region for the ZaZiBoNa process (21, 26). Having a limited number of assessors with adequate skills leads to delayed timelines in medicine approval. There is a general scarcity of pharmaceutical professionals in most LMICs caused by low salaries, “brain drain,” and lack of career structure (28). MCAZ has done well by participating in several collaborative partnerships to capacitate its regulatory officers, but to retain them and have the maximum gain from all the training invested in its staff, MCAZ needs to ensure its staff is well renumerated to ensure the sustainability of these great partnerships.

Medicine approval is a complex process that is essential to ensure medicines registered in a country are of good quality, safe, and efficacious. The process can be time-consuming and result in delayed access to essential medicines for affected or at-risk populations. MCAZ has been able to register two HIV PrEP products, namely, DVR and CAB-LA, in a shorter time frame than other LMICs because of collaborations with other NMRAs and clinical researchers in the region and better-resourced medicine regulatory authorities from high-income countries. These collaborations have built the capacity of MCAZ regulatory officers and avoided duplication of effort, enabling them to efficiently review submissions promptly.

## Data Availability

The original contributions presented in the study are included in the article/Supplementary material; further inquiries can be directed to the corresponding author.
